# The Efficacy of Parathyroid Hormone Analogues in Combination With Bisphosphonates for the Treatment of Osteoporosis: A Meta-Analysis of Randomized Controlled Trials

**DOI:** 10.1097/MD.0000000000001156

**Published:** 2015-09-25

**Authors:** Wan Li, Wenjian Chen, Yang Lin

**Affiliations:** From the Department of Ophthalmology, Union Hospital (WL); and Department of Orthopaedic Surgery, Tongji Hospital, Tongji Medical College, Huazhong University of Science and Technology, Wuhan, Hubei Province, China (WC, YL).

## Abstract

Parathyroid hormone (PTH) analogues increase bone strength primarily by stimulating bone formation, whereas antiresorptive drugs (bisphosphonates) reduce bone resorption. Therefore, some studies have been designed to test the hypothesis that the concurrent administration of the 2 agents would increase bone density more than the use of either one alone. This meta-analysis aimed to determine whether combining PTH analogues with bisphosphonates would be superior to PTH alone.

Electronic databases were searched to identify relevant publications up to March, 2014. Randomized controlled trials (RCTs) comparing PTH analogues combined bisphosphonates with PTH for osteoporosis were analyzed. According to the Cochrane Handbook for systematic Reviews of Interventions 5.2, we identified eligible studies, evaluated the methodological quality, and abstracted relevant data.

Totally 7 studies involving 641 patients were included for meta-analysis. The pooled data showed that there were no significant differences in the percent change of spine BMD (MD_1-year_ = −0.97, 95% CI −2.81 to 0.86, *P* = 0.30; MD_2-year_ =  − 0.57, 95% CI −5.01 to 6.14, *P* = 0.84), femoral neck BMD (MD_1-year_ = 0.60, 95% CI −0.91 to 2.10, *P* = 0.44; MD_2-year_ = −0.73, 95% CI −4.97 to 3.51, *P* = 0.74), the risk of vertebral fracture (risk ratio [RR] = 1.27; 95% CI 0.29–5.57; *P* = 0.75), and the risk of nonvertebral fracture (RR = 0.97; 95% CI 0.40–2.35; *P* = 0.95) between the 2 groups, whereas combination group improves the percent change of hip BMD at 1 year (MD = 1.16, 95% CI 0.56–1.76; *P* < 0.01) than PTH analogues group.

Our results showed that there was no evidence for the superiority of combination therapy, although significant change was found for hip BMD at 1 year in combination group. Further large multicenter randomized controlled trials are still needed to investigate the efficacy of combination therapy.

## INTRODUCTION

Osteoporosis is a major public health problem that worsens the quality of life in the aging populations worldwide,^1^ and has become one of the most prevalent, debilitating, and important chronic diseases.^2^ The most direct consequence of osteoporosis is the increased incidence of fractures, which is associated with considerable morbidity and mortality in older adults.^3^ In the United States, it was estimated that the annual cost of fractures related to osteoporosis in 2005 was $16.9 billion, with the expectation that this figure would rise to $25.3 billion by 2025.^4^

To prevent fragility fractures in patients with osteoporosis, a range of pharmacological therapies are available, most of which includes either antiresortives, which inhibit bone resorption, enhanced in postmenopausal osteoporosis, or bone-forming agents that restore bone mass.^5^ These include bisphosphonate therapy,^6^ estrogen and combined hormone replacement therapy (HRT),^7^ selective estrogen receptor modulators (SERM)^8^ and calcitonin,^9^ along with calcium and vitamin D.^10^ Besides, parathyroid hormone (PTH) peptide PTH [1–34] (teriparatide) and the full-length molecule PTH [1–84], which have anabolic skeletal effects, are also widely used for osteoporosis treatment.^11,12^

The combination therapy with antiresorptive and osteoanabolic drugs is based upon the hypothesis that if bone formation is stimulated by an osteoanabolic agent while bone resorption is inhibited by an antiresorptive agent (such as bisphosphonate), the combination might lead to better results than monotherapy with either agent alone. Several studies^13–19^ have been designed to investigate the combination therapy with PTH analogues and bisphosphonates. Some studies^13,15,18^ found combination therapy could improve the level of bone mineral density (BMD) than single therapy whereas others studies^14,16,17,19^ reported no differences between combination therapy and single therapy. To date, the conclusions among studies are still controversial.

Therefore, we designed this meta-analysis of all available randomized controlled trials (RCTs) to quantitatively investigate whether combination therapy is superior to PTH analogues alone for the treatment of osteoporosis.

## METHODS

Our study was conducted according to the Preferred Reporting Iterms for Systematic reviews and Meta-analyses (PRISMA) statement.^20^

### Literature Search

A systematic literature search was performed to identify relevant RCTs published up to January 2015. Pubmed, Embase, Cochrane Library Clinical Trials databases and Web of Science were searched according to predefined search strategy with terms relevant to osteoporosis, bisphosphonates, alendronate, ibandronate, risedronate, zoledronic acid, teriparatide, PTH analogues, and RCTs. Meanwhile, the references of the retrieved articles and relative review were also identified. Two reviewers independently completed this search and disputes were resolved by other authors.

### Inclusion Criteria

To satisfy the conditions for inclusion, the studies had to meet the following criteria: a prospective RCT comparing PTH analogues combined with bisphosphonate versus PTH analogues alone; a minimum of 12-month clinical follow-up; patients aged 18 years or older were eligible along with studies in patients with secondary (for example, glucocorticoid-induced) osteoporosis; at a minimum one outcome postoperatively. Two reviewers assessed the articles independently to determine whether they met the inclusion criteria. Disagreements between reviewers were resolved by consensus.

The outcomes we assessed included mean change in the BMD of spine, hip, and femoral neck, and the risk of vertebral fracture and nonvertebral fracture. If several publications reported on the same trial data, we chose the report with the longest follow-up and the most detailed information of outcome. Two of the authors reviewed the studies independently. We decided to study eligibility and data extraction by consensus. Interrater reliability was assessed, and no deviations in the studies retrieved.

### Data Extraction and Quality Appraisal

The following data were abstracted by 2 independent authors onto standardized forms: first author, publication year, study design, age of participants, sample size, gender ratio, details of intervention, and the follow-up interval. Disagreements were resolved through discussion with the participation of other authors if necessary.

Two authors read the full articles and assessed the quality of included studies independently according to the Cochrane Handbook for Systematic Reviews of Interventions, Version 5.2.^21^ Quality ratings were made according to the Cochrane Collaboration guidelines: random sequence generation (selection bias), allocation concealment (selection bias), blinding of participants and personnel (performance bias), blinding of outcome assessment (detection bias), incomplete outcome data (attrition bias), selective reporting (reporting bias), and other bias. All discrepancies were resolved by consensus.

We assessed strength of evidence for each major outcome according to the Grading of Recommendations Assessment, Development, and Evaluation (GRADE) criteria.^22^ Both investigators made judgments on risk for bias, precision, consistency, directness, and likelihood of publication bias.

### Statistical Analysis

The risk ratio (RR) and its corresponding 95% confidence interval (CI) were calculated for dichotomous data, and the measurement data were summarized using the mean difference (MD) and its corresponding 95% CI. Statistical heterogeneity was quantified using the *I*^2^ statistic. The random-effects model was used if there was heterogeneity between studies (*I*^2^ >50%); otherwise, the fixed-effects model was used (*I*^2^ <50%). Statistical analyses were performed using Review Manager 5.2 software (RevMan 5, The Cochrane Collaboration, Oxford, UK), with *P* value <0.01 being considered as statistically significant.^21^ This is a meta-analysis of literatures, so ethical approval was not necessary for our research.

## RESULTS

### Search Result

**FIGURE 1:**
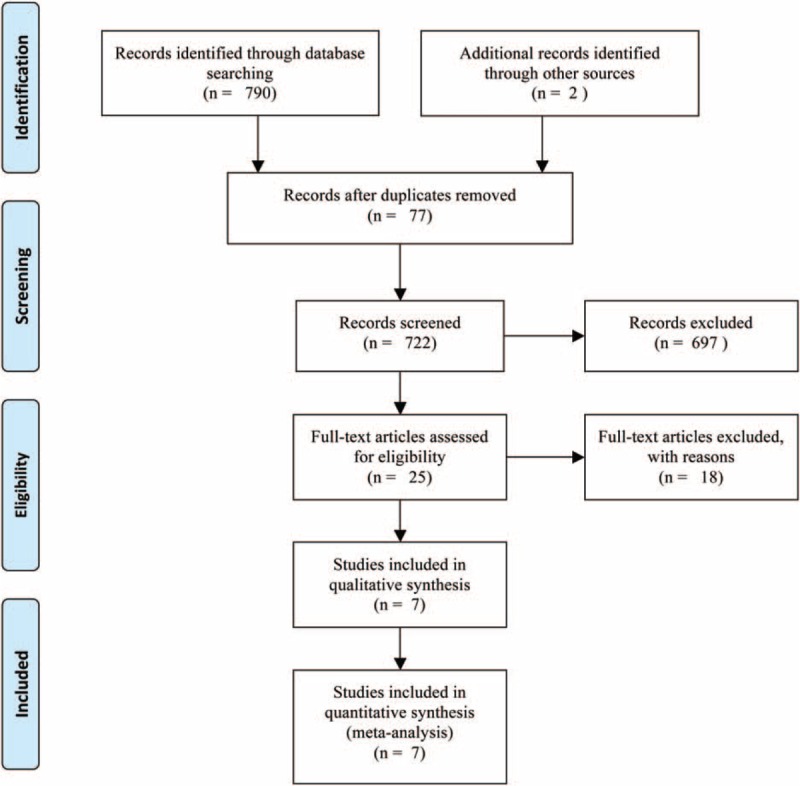
The flow chart of literature screening.

The literature search strategy identified 792 publications across electronic databases and reference of included studies. After exclusion of duplicates and the initial screen of titles and abstracts articles, we obtained 25 full articles of potentially relevant studies. After full-text reviews, 7 studies^13–19^ from 6 RCTs with 641 patients were included for the eventually analysis. Figure 1 shows study selection process and results from the literature search.

### Characteristics and Methodological Quality of Included Studies

Table 1 presents the characteristics of the included trials. In aggregate, the studies involved 641 individuals, including 334 individuals in combination group and 307 patients in the PTH analogues group. The number of patients included in these RCTs ranged from 19 to 275. In those included studies, the period of follow-up ranged from 12 to 30 months. PTH analogues were used in 4 studies^16–19^ and teriparatide was used in 3 studies.^13–15^ The dose of PTH analogues used ranged from 20 to 100 μg, the dose of alendronate, risedronate, and zoledronic acid were 10 to 70 mg, 5 mg, 35 mg, separately. All participants received oral calcium (500–1200 mg) and vitamin D (400 IU) supplements daily. The baseline parameters of included studies were comparable between the combination group and single-drug group (Table 1).

**TABLE 1:**
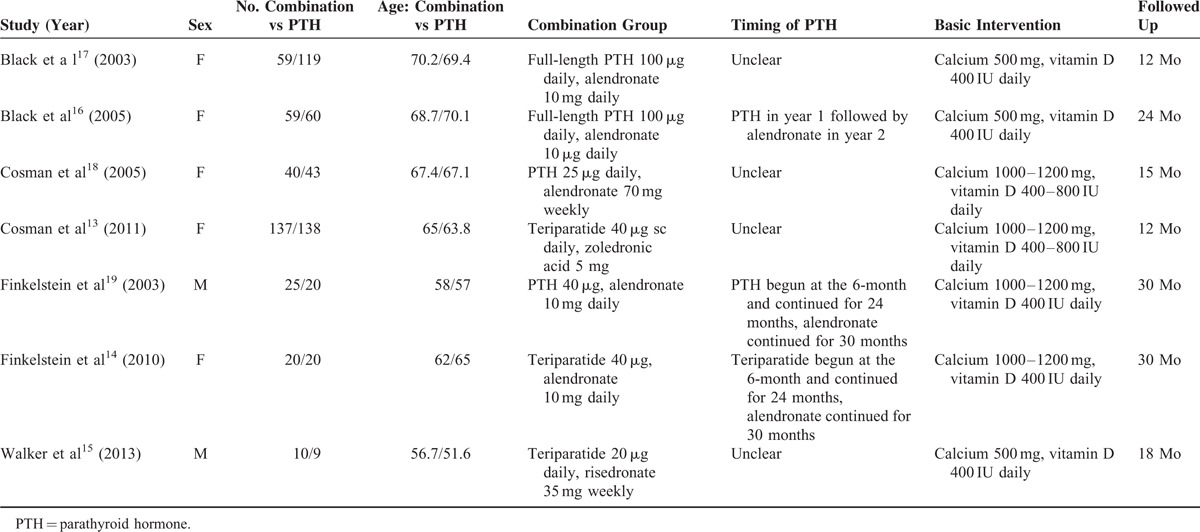
Characteristics of Included Studies

Five studies reported adequate generation of allocation sequence except 2 studies,^16,17^ and 5 studies^13,15–17,19,23^ performed the blinding to participants or assessors, whereas none of included studies reported the allocation concealment. The methodological quality of included studies was presented in Table 2.

**TABLE 2:**
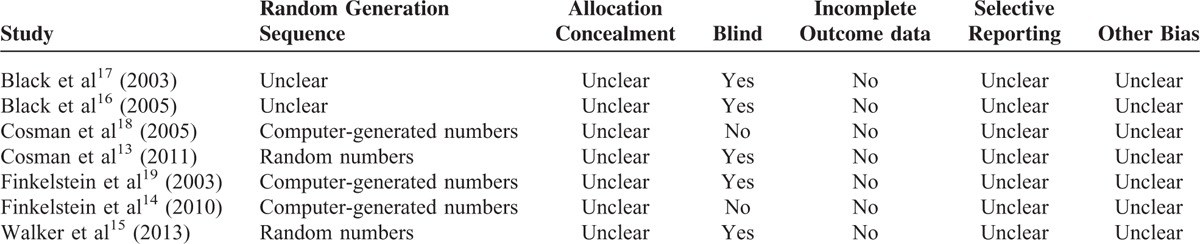
Risk of Bias in Included Studies

### Effect of Intervention

#### Mean Percent Change in Spine BMD

Five studies^13–15,17,19^ with 565 patients reported the outcome of spine BMD. The meta-analysis showed that there were no significantly statistical differences in spine BMD at either 1 year (MD = −0.97; 95% CI −2.81 to 0.86; *P* = 0.30) or 2 year (MD = −0.57; 95% CI −5.01 to 6.14; *P* = 0.84) follow-up between combination group and PTH analogues group.

#### Mean Percent Change in Hip BMD

The data of hip BMD were available in all the included studies^13–19^ involving 641 patients. Meta-analysis showed that combination group could significantly increase the hip BMD at 1 year (MD = 1.16; 95% CI 0.56–1.76; *P* < 0.01) than PTH analogues group, whereas there was no significant difference at 2-year (MD = 1.38; 95% CI −0.76 to 3.51; *P* = 0.21) follow-up between groups.

#### Mean Percent Change in Femoral Neck BMD

Information on the mean percent change in femoral neck BMD was provided in 5 studies^13–15,17,19^ with a total of 565 patients. The outcome of meta-analysis demonstrated that there were no significantly statistical differences in femoral neck BMD at 1 year (MD = 0.60; 95% CI −0.91 to 2.10; *P* = 0.44) and 2-year (MD = −0.73; 95% CI −4.97 to 3.51; *P* = 0.74) follow-up between the 2 groups.

### Vertebral Fractures

**FIGURE 2:**
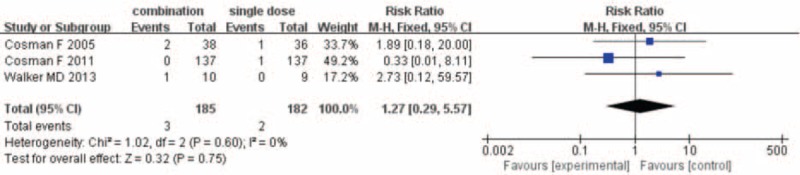
Result of meta-analysis of vertebral fracture.

Data of vertebral fracture mentioned by 3 studies^13,15,18^ involving 367 patients were pooled together for meta-analysis. Vertebral fracture occurred in 3 of 185 (1.6%) patients in the combination group and 2 of 182 patients (1.1%) in the PTH analogues group. As depicted in Table 3, there was no evidence of statistical heterogeneity between all studies (*I*^2^ = 0%). The resulting meta-analysis showed that there was no significant difference in vertebral fracture between groups (RR = 1.27; 95% CI 0.29–5.57; P = 0.75) (Fig. 2).

**TABLE 3:**
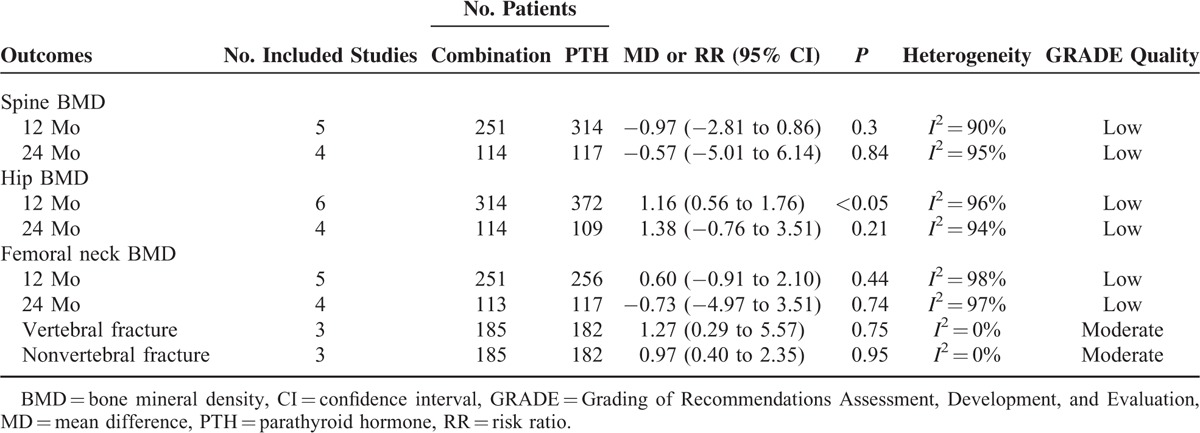
The GRADE Evidence Quality for Each Outcome

### Nonvertebral Fractures

**FIGURE 3:**
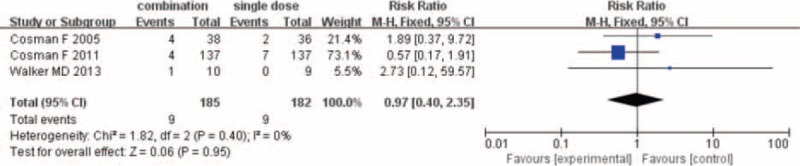
Result of meta-analysis of nonvertebral fracture.

The number of nonvertebral fracture was provided by 3 studies^13,15,18^ for meta-analysis. Nonvertebral fracture occurred in 9 of 185 (4.4%) patients in the combination group and 9 of 182 patients (4.9%) in the single-drug group. The fixed-effects model was used because statistical heterogeneity was not found between studies (*I*^2^ = 0%). The resulting meta-analysis revealed no statistically significant difference in nonvertebral fracture between combination group and PTH analogues group (RR = 0.97; 95% CI 0.40–2.35; *P* = 0.95) (Fig. 3).

### The Level of Evidence of Main Outcomes

The level of evidence of main outcomes of our meta-analysis was assessed using GRADE system. The level of evidence of vertebral fracture and nonvertebral fracture was moderate, whereas the level of evidence of mean change in spine BMD, hip BMD, and femoral neck BMD was low.

## DISCUSSION

### Summary of Main Findings

The most important finding of our meta-analysis was that compared with PTH analogues therapy alone, PTH combined with bisphosphonates (combination group) increases the hip BMD at 1 year follow-up, whereas there were no significant differences in the change of spine or femoral neck BMD and the risk of vertebral or non-vertebral fracture between combination group and PTH analogues group. The evidence grade of main outcomes in present review accessed by the GRADE system was deemed to be at moderate level for vertebral fracture and nonvertebral fracture, whereas the outcomes of spine, hip, and femoral neck BMD were grading low.

Nowadays, antiresorptives and anabolic agents are among the most popular drugs used for the treatment of osteoporosis. Antiresorptive drugs reduce the activation frequency, acting mostly on osteoclast and only indirectly on osteoblast activity, with the final slight gain in trabecular bone mass.^24^ As one of the antiresorptives drugs, bisphosphonates (risedronate, alendronate and zoledronic acid) are the most prescribed for treatment and prevention of osteoporosis.^25^ Unlike antiresorptives, anabolic therapies directly stimulate bone formation through activation of bone modeling, independently of resorption activity^26^ and have been employed for osteoporosis treatment recently.^27^ At present, PTH analogues or its fully active fragment PTH 1–34 (teriparatide) are the only available anabolic drugs for the treatment of osteoporosis.

Could we combine the antiresorptives (reduce the bone resorption) drugs and anabolic agents (stimulate bone formation) for osteoporosis treatments, other than the use of either agent alone? To test this hypothesis, some well-designed studies have investigated the effects of PTH analogues combined with bisphosphonates versus PTH analogues alone in patients with osteoporosis. In 2003, Black et al^17^ and Finkelstein et al^19^ firstly reported their results of combination therapy in patients with osteoporosis. In the trial by Black et al,^17^ 238 postmenopausal women with low BMD were randomly assigned to daily treatment with single drug (PTH analogues, 100 μg or alendronate, 10 mg) or combination therapy and they demonstrated that there was no significant difference in the increase of BMD between the combination-therapy group and single-drug group. While in the study by Finkelstein et al,^19^ authors randomly assigned 83 men who had low BMD to receive single drug (alendronate, 10 mg daily or PTH analogues, 40 μg subcutaneously daily) or combination therapy, and found that combination therapy did not increase the spine or femoral neck BMD compared with PTH. Finkelstein et al concluded that alendronate impaired the ability of PTH analogues to stimulate new bone formation in men. However, in 2011, Cosman et al^13^ randomized 412 postmenopausal women to receive single drug (zoledronic acid, 5 mg or teriparatide, 20 μg daily) or combination therapy (zoledronic acid 5 mg plus daily subcutaneous teriparatide 20 μg) and reported that combination group could significantly increase the spine BMD and hip BMD compared with single-drug group. Currently, the available data of published trials provided conflicting results and it was necessary to pool these results of different studies to investigate whether combination therapy was superior to single drug.

The primary outcomes of our study were the change of BMD in spine, hip, and femoral neck. By pooling 7 RCTs involving 641 patients, our study found that compared with PTH analogues alone, there was no advantage to combination therapy, but it was associated with greater increases in change of hip BMD at 1-year follow-up with combination therapy than PTH analogues alone. These findings were consistent with previous studies.^13,15,17^ In addition, the level of evidence of these outcomes was grading low. The reasons of downgrading the evidence are the high heterogeneity among studies and low quality of included studies.

Another important outcome was the risk of fracture. Our meta-analysis showed that compared with PTH analogues alone, combination group could not reduce the risk of vertebral fracture and nonvertebral fracture. The fracture risk estimates did not display heterogeneity, although the level of evidence was grading moderate, but the number of studies was limited. Only 3 included studies^13,15,18^ involving 731 patients were included, and future long-term and large-sample studies were still needed to evaluate the risk of fracture.

### Strength and Limitation of This Meta-Analysis

There are some strengths in our meta-analysis. This study was based on several prospective randomized studies from various populations. Besides, this study was strictly conducted in according with PRIMA guideline and the level of evidence of outcomes was assessed by the GRADE system.

There were, however, several limitations of this meta-analysis. First, the methodological quality of included studies was low. For instance, none of included studies reported the allocation concealment, which may limit the reliability of the pooled results; second, there is still great heterogeneity in outcomes of spine, hip, and femoral neck BMD, which indicated that other factors should have been taken into account in the analysis. One of the main cause is the different dose of PTH analogues (20–100 μg) or different bisphosphonates (risedronate, alendronate, and zoledronic acid). Third, the adverse events were not well studied for the limited data of included studies; future studies should investigate the both short- and long-term adverse events of combination therapy. Finally, the number of the trials included was small; therefore, large, well-designed, and multicenter RCTs are still needed.

## CONCLUSIONS

Based on 7 studies involving 641 patients, we concluded that there was no evidence for the superiority of combination therapy, although significant change was found for hip BMD at 1 year in combination group. Further large multicenter RCTs are still need to investigate the efficacy of combination therapy.
